# Gender differences in nerve regeneration after sciatic nerve injury and repair in healthy and in type 2 diabetic Goto-Kakizaki rats

**DOI:** 10.1186/1471-2202-15-107

**Published:** 2014-09-13

**Authors:** Lena Stenberg, Lars B Dahlin

**Affiliations:** Department of Clinical Sciences - Hand Surgery, Lund University, Skane University Hospital, Malmö, Sweden

**Keywords:** Nerve regeneration, Nerve repair, Neuropathy, Diabetes, Gender differences, Schwann cells

## Abstract

**Background:**

In view of the global increase in diabetes, and the fact that recent findings indicate that diabetic neuropathy is more frequently seen in males, it is crucial to evaluate any gender differences in nerve regeneration in diabetes. Our aim was to evaluate in short-term experiments gender dissimilarities in axonal outgrowth in healthy and in genetically developed type 2 diabetic Goto-Kakizaki (GK) rats, and also to investigate the connection between activated (i.e. ATF-3, Activating Transcription Factor 3) and apoptotic (cleaved caspase 3) Schwann cells after sciatic nerve injury and repair. Female and male diabetic GK rats, spontaneously developing type 2 diabetes, were compared with corresponding healthy Wistar rats. The sciatic nerve was transected and instantly repaired. After six days the nerve was harvested to measure axonal outgrowth (i.e. neurofilament staining), and to quantify the number of ATF-3 (i.e. activated) and cleaved caspase 3 (i.e. apoptotic) stained Schwann cells using immunohistochemistry.

**Results:**

Axonal outgrowth was generally longer in male than in female rats and also longer in healthy than in diabetic rats. Differences were observed in the number of activated Schwann cells both in the distal nerve segment and close to the lesion site. In particular the female diabetic rats had a lower number. There were no gender differences in number of cleaved caspase 3 stained Schwann cells, but rats with diabetes exhibited more (such cleaved caspase 3 stained Schwann) cells both at the lesion site and in the distal part of the sciatic nerve. Axonal outgrowth correlated with the number of ATF3 stained Schwann cells, but not with blood glucose levels or the cleaved caspase 3 stained Schwann cells. However, the number of cleaved caspase 3 stained Schwann cells correlated with the blood glucose level.

**Conclusions:**

We conclude that there are gender differences in nerve regeneration in healthy rats and in type 2 diabetic GK rats.

## Background

Neuropathy, which is a general complication in both type 1 and type 2 diabetes, may cause serious problems for patients, particularly male patients with type 2 diabetes [1]. Male patients also seem to develop neuropathy earlier than female patients [1, 2]. The mechanisms behind the development of neuropathy are complex and not fully understood, nor why male patients express neuronal complications earlier than female patients. The gender differences are also relevant in traumatic nerve injuries since such injuries more frequently affect males.

Earlier studies have shown that neuroactive hormones or steroids (affecting the nervous systems), such as testosterone and progesterone, play an active role in nerve regeneration and that the levels of neuroactive steroids differ in male and female rats, among both healthy and diabetic rats [3–5]. Recent findings indicate that axonal outgrowth six days after nerve injury and repair does not differ between healthy and diabetic BB (i.e. genetically developed and resembling type 1 diabetes; [6]) (female) rats. However, both numbers of activated and apoptotic Schwann cells, which are present in the distal nerve segment, were significantly higher in female diabetic BB rats than in female healthy rats [6]. Interestingly, a positive correlation between axonal outgrowth and Schwann cell activation (i.e. ATF 3 staining) has been reported, indicating the importance of Schwann cells for nerve regeneration and that the time point for nerve repair is crucial for axonal outgrowth [7–9]. Clarification of the nerve regeneration process from a gender perspective as well as in relation to a rat model with genetically developed diabetes similar to type 2, in contrast to the streptozotocin-induced diabetes model [10], is needed. This is relevant in view of the global increase in the number of patients with type 2 diabetes, where neuropathy is often present, and the fact that males are frequently affected by traumatic nerve injuries, particularly in the upper extremity, which require nerve repair or reconstruction. Thus, our aims were two-fold; to investigate any gender differences in nerve regeneration after nerve injury and repair in healthy rats from a short-term perspective and to explore whether the process is different in genetically developed type 2 Goto-Kakizaki diabetic rats. In conclusion, we observed differences between healthy and diabetic rats, using an appropriate diabetic animal model, as well as between male and female rats, with interesting correlations.

## Results

### Fasting blood glucose and weight increase

Pre- and post-operative blood glucose levels were significantly higher (Fisher’s method; p < 0.0001) in diabetic than in healthy rats and were also significantly higher p < 0.0001) in male than in female rats (Table 1). There were no statistical differences between the groups concerning increase in weight during the regeneration period (Table 1).

**Table 1:** **Nerve regeneration after nerve injury and repair in healthy and diabetic rats**

	Healthy male	Healthy female	Diabetes male	Diabetes female	p-values (KW ^a^)	Fisher’s method ^b^	
	(n = 10)	(n = 10)	(n = 10)	(n = 10)		Male/female	Healthy/Diabetes
**Axonal outgrowth (neurofilaments, mm)**	7.0 (6.8-7.3)	6.3 (6.1-6.4)	6.9 (6.7-7.1)	5.8 (5.1-6.2)	**0.0001**	**<0.0001**	**0.02**
**ATF-3 (% of total) SNL**	17.8 (17.6-18.4)	17.6 (16.7-18.0)	19.8 (19.1-21.1)	16.4 (15.7-17.3)	**0.0001**	**0.0001**	**0.001**
**ATF-3 (% of total) SND**	19.2 (17.7-19.6)	19.5 (18.9-20.1)	18.7 (16.9-20.1)	15.2 (14.4-15.7)	**0.0001**	**0.0003**	**0.001**
**Cleaved caspase-3 (% of total) SNL**	5.2 (2.6-7.8)	3.1 (2.9-3.4)	9.6 (8.7-11.6)	7.0 (6.4-10.1)	**0.0001**	0.31	**<0.0001**
**Cleaved caspase-3 (% of total) SND**	2.7 (2.2-4.0)	2.5 (2.4-2.7)	6.4 (4.5-7.1)	5.8 (4.3-6.1)	**0.0001**	0.39	**<0.0001**
**Total cell number (mm** ^**2**^ **) SNL**	1137 (1127-1142)	1050 (1027-1066)	1073 (1046-1103)	1089 (1059-1098)	**0.0001**	**0.0007**	**0.0001**
**Total cell number (mm** ^**2**^ **) SND**	1137 (1122-1152)	1112 (1068-1147)	1053 (1019-1091)	1093 (1045-1102)	**0.0001**	0.07	**<0.0001**
**Preoperative B-glucose (mmol/l)**	4.6 (4.3-4.9)	3.90 (3.8-4.3)	15.0 (13.3-17.2)	10.3 (9.7-11.7)	**0.0001**	**<0.0001**	**<0.0001**
**Weight increase (%)**	2.2 (0.7-2.7)	2.4 (1.3-4.8)	2.0 (1.2-2.6)	2.6 (2.0-3.2)	0.34	**-**	**-**

Values are median 25^th^ (Q1) -75^th^ (Q3) percentiles. ^a^KW = Kruskal-Wallis. ^b^Fisher’s method for independent samples based on the chi square distribution. *SNL* = Lesion site. *SND* = Distal nerve segment. *Total cell* = DAPI stained cells. P-values in bold is significantly different at least at the 0.05 level.

### Length of axonal outgrowth

**Figure 1:**
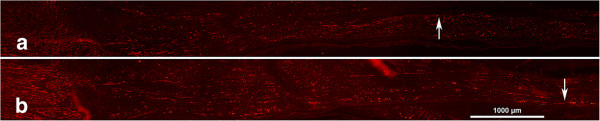
**Immunohistochemistry of neurofilament staining.** Immunohistochemistry of staining for neurofilament proteins in female **(a)** and male **(b)** GK rats. The arrow indicates tip of the outgrowing axons, where the nerve repairs are in the left part of the panel. Length of bar = 1000 μm.

Statistical differences were found between male and female rats (p < 0.0001; Fisher’s test; Table 1) with longer outgrowth (Figure 1) in male rats (p < 0.0001 for both healthy and diabetic rats, MW). There were also distinctions between healthy and diabetic rats (p = 0.02; Fisher’s test), where axonal outgrowth was much longer in female healthy rats than in female diabetic rats (p = 0.004, MW, Table 1), while no difference was observed between healthy and diabetic male rats (p = 0.68, MW).

### ATF-3 stained Schwann cells at the site of lesion

**Figure 2:**
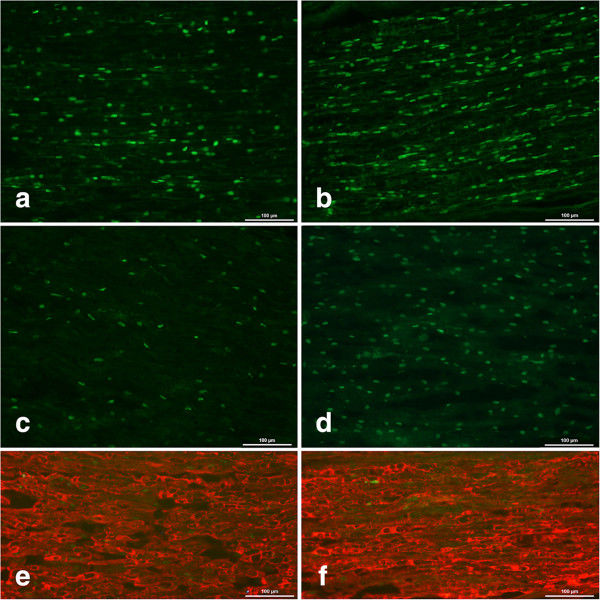
**Immunohistochemistry of ATF3 and cleaved caspase 3 stained Schwann cells.** Immunohistochemistry of ATF3 stained Schwann cells in Wistar **(a)** and GK **(b)** male rats as well as cleaved caspase 3 stained cells in Wistar **(c)** and GK **(d)** male rats; all at the lesion site. Double staining of ATF 3 and S-100 **(e)** as well as cleaved caspase 3 and S-100 **(f)**, at the lesion site in a male GK rat. Length of bar = 100 μm.

Only those cells with a Schwann cell shape were counted as ATF-3 stained Schwann cells (Figure 2a,c-d). ATF-3 stained Schwann cells differed (p = 0.0001; KW) at the lesion site. The Fisher’s test showed a statistical difference (p = 0.0001) between male and female rats and also between healthy and diabetic rats (p = 0.001). The number of ATF-3 stained Schwann cells was higher (p = 0.001 MW) in diabetic rats than in healthy male rats. The numbers were also higher (p = 0.0001, MW) in diabetic male rats than in diabetic female rats. On the contralateral side (i.e. uninjured side), only a few ATF-3 stained Schwann cells were found.

### ATF-3 stained Schwann cells in the distal nerve segment

Again, only cells with the shape of a Schwann cell were counted as ATF-3 stained Schwann cells. ATF-3 stained Schwann cells differed (p = 0.0001; KW) in the distal segment. The Fisher’s test demonstrated significant differences between male and female rats (p = 0.0003) as well as between healthy and diabetic rats (p = 0.001). Higher numbers of ATF-3 stained Schwann cells (p = 0.0001, MW) were found in diabetic male rats than in diabetic female rats and in healthy female rats than in diabetic female rats (p = 0.0001, MW). Again, few ATF-3 stained Schwann cells were found on the contralateral side.

### Cleaved caspase-3 stained Schwann cells at the lesion site

Generally, there were differences (p = 0.0001; KW) regarding numbers of cleaved caspase 3 stained Schwann cells at the lesion site in the sciatic nerve (Figure 2b,e-f). There were mostly no gender differences in the number of cleaved caspase 3 stained Schwann cells at the lesion site (p = 0.31, Fisher’s test) among male and female rats. However, a significant difference was found between healthy and diabetic rats (p < 0.0001, Fisher’s test). The number of stained cleaved caspase 3 Schwann cells was statistically higher (p = 0.0001, MW) in diabetic male rats than in healthy male rats and also in diabetic female rats than in healthy female rats (p = 0.0001 MW). Single cleaved caspase 3 stained Schwann cells were found on the contralateral side.

### Cleaved caspase 3 stained Schwann cells in the distal nerve segment

There were generally differences (p = 0.0001; KW) regarding cleaved caspase 3 stained Schwann cells in the distal nerve segment. Furthermore, Fisher’s test showed no significant differences between male and female rats (p = 0.39), but a significant difference between healthy and diabetic rats (p < 0.0001). A significantly higher number of cleaved caspase-3 stained Schwann cells (P = 0.0001, MW) was found in diabetic female rats than in healthy female rats. There were also significantly higher numbers in diabetic male rats (p = 0.0001, MW) than in healthy male rats. Again, single cleaved caspase 3 stained Schwann cells were found on the contralateral side.

### Total number of DAPI stained cells at the lesion site

Kruskal-Wallis showed differences (p = 0.0001) with respect to total number of DAPI stained cells at the lesion site. There were significant differences in the total number of DAPI stained cells between male and female rats (p = 0.0007, Fisher’s test; Table 1) and between healthy and diabetic rats (p = 0.0001). The total number of DAPI stained cells at the lesion site was higher in the healthy male rats than in healthy female rats (p = 0.0001, MW) and in healthy male rats than in diabetic male rats (p = 0.0001, MW).

### Total number of DAPI stained cells in the distal nerve segment

Kruskal-Wallis showed differences (p = 0.0001) in the total number of DAPI stained cells in the distal nerve segment. Significant differences were observed between healthy and diabetic rats (p < 0.0001, Fisher’s test), but not between male and female rats (p = 0.07; Fisher’s test). There was a significantly higher number of total DAPI stained cells in healthy male rats than in diabetic male rats (p = 0.0001, MW).

### Correlation

#### All rats pooled

**Figure 3:**
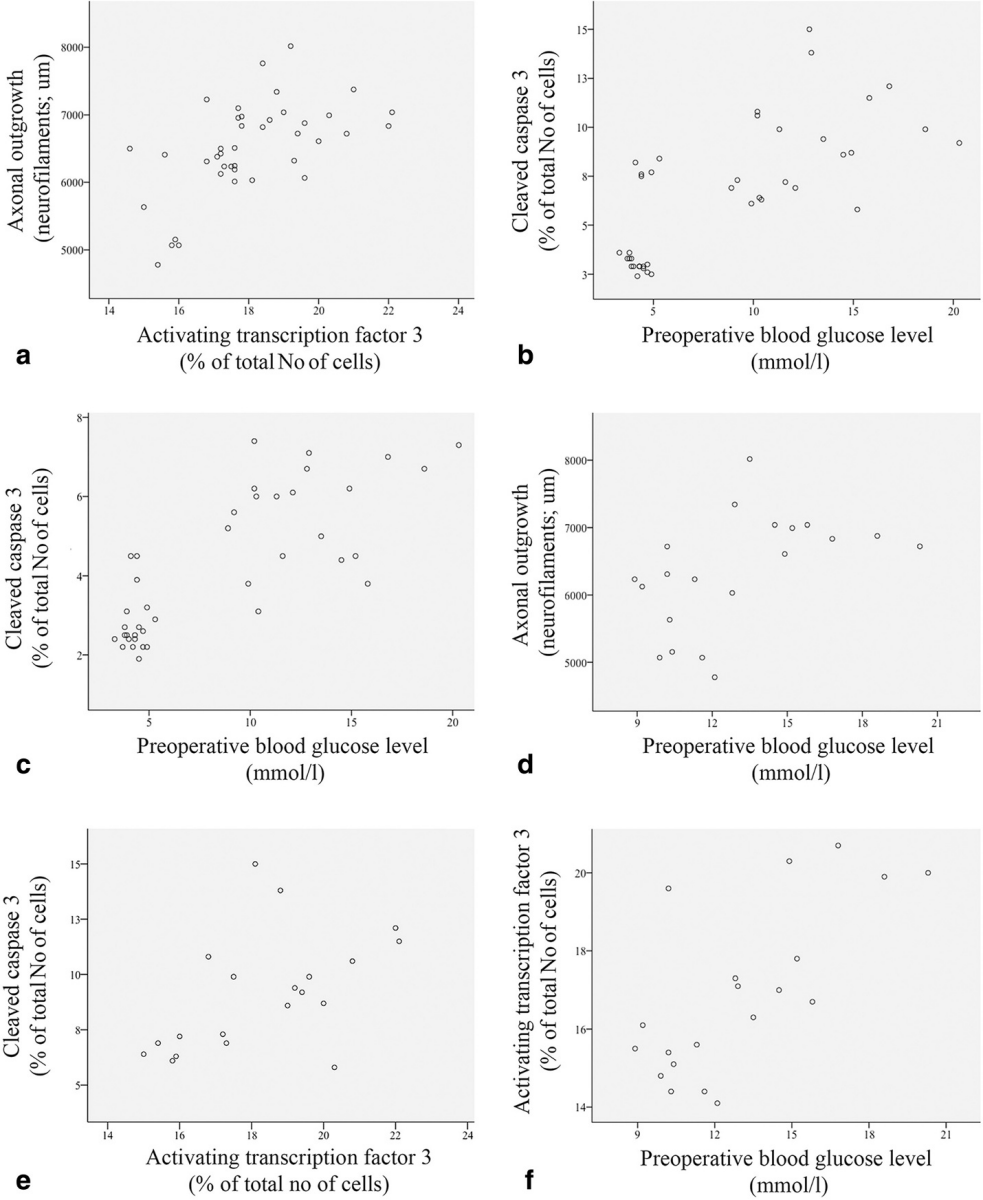
**Scatter plots from all rats (a-c) and rats with diabetes (d-f).** Scatter plots from all rats pooled **(a-c)** with numbers of ATF-3 stained cells (lesion site) and axonal outgrowth (**a**; rho = 0.59, p = 0.0001), preoperative blood glucose level and cleaved caspase 3 stained cells (**b**, lesion site; rho = 0.69, p = 0.0001; **c**. in distal nerve segment; rho = 0.74, p = 0.0001). Scatter plots from diabetic rats **(d-f)** with preoperative blood glucose levels and axonal outgrowth (d, rho = 0.60, p = 0.005; **d**), number of cleaved caspase 3 and ATF-3 stained cells (**e**, lesion site; rho = 0.51, p = 0.02) and preoperative blood glucose level and number of ATF-3 stained cells (**f**, distal nerve segment; rho = 0.66, p = 0.001).

The Spearman correlation test showed a significant positive correlation between numbers of ATF-3 stained Schwann cells at the lesion site and axonal outgrowth (rho = 0.59, p = 0.0001; Figure 3a), but blood glucose levels did not correlate with axonal outgrowth (p = 0.39) when all rats were pooled. Preoperative blood glucose level and cleaved caspase 3 stained Schwann cells at the lesion site were also tested for correlation and found to be positively correlated (rho = 0.69, p = 0.0001; Figure 3b). A weaker correlation (rho = 0.32, 0 = 0.046) was found between preoperative blood glucose and numbers of ATF-3 stained Schwann cells at the lesion site. While there was no significant correlation between the numbers of cleaved caspase 3 stained Schwann cells and axonal outgrowth (p = 0.24), they correlated weakly with the number of ATF-3 stained Schwann cells (rho = 0.40, p = 0.01).

In the distal nerve segment, when all rats were pooled, the preoperative blood glucose levels positively correlated with the number of cleaved caspase 3 stained Schwann cells (rho = 0.74, p = 0.0001, Figure 3c), but the blood glucose levels did not correlate with the numbers of ATF 3 stained Schwann cells (p = 0.11). The weight increase in the rats did not influence any of the variables.

#### Diabetic rats

If the diabetic animals were analysed separately, there was a positive correlation between preoperative blood glucose levels, and axonal outgrowth (rho = 0.60, p = 0.005; Figure 3d) and ATF-3 stained Schwann cells at the site of the lesion (rho = 0.67, p = 0.001) as well as between the number of ATF-3 and cleaved caspase 3 stained Schwann cells at the lesion site (rho = 0.51, p = 0.02; Figure 3e). Furthermore, in the diabetic rats the preoperative blood glucose levels positively correlated with the number of ATF-3 stained Schwann cells in the distal nerve segment (rho = 0.66, p = 0.001, Figure 3f).

#### Healthy rats

The blood glucose levels in healthy rats positively correlated with axonal outgrowth (rho = 0.60, p = 0.005).

## Discussion

**Figure 4:**
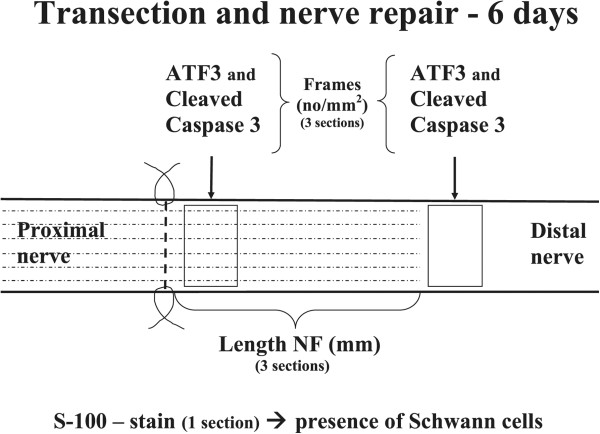
**Schematic drawing of the experimental set up.** The sciatic nerve was transected and immediately repaired. Axonal outgrowth was evaluated by measuring the length of neurofilaments (NF). The numbers of activated (Activating Transcription Factor 3; ATF3) and apoptotic (cleaved caspase 3) Schwann cells were measured just distal to the lesion site (i.e. axons presented) and in the distal nerve segment (distal to the most distal parts of the neurofilament stained axons).

We examined whether any gender differences between female and male rats, regarding axonal outgrowth, were present. In particular, we analysed how a high blood glucose level in a diabetic GK rat model, resembling type 2 diabetes, influenced the axonal outgrowth. The published literature in the field of nerve regeneration in diabetes is limited and studies almost exclusively use streptozotocin-induced diabetes in rats in which blood glucose levels are high (see e.g. [11–14]). The ability of axons to regenerate in relation to the state of the Schwann cells may also be an important point in relation to moderate blood glucose levels and in view of the global increase of diabetes, particularly bearing in mind its most common complication, i.e. neuropathy. We used genetically developed diabetic GK rats as a model for type 2 diabetes, which is a valid model and also a gentle alternative to the diabetic BB (BioBreeding) rats [6] and the frequently used rats with streptozotocin-(STZ)induced diabetes. The similarity with human beta cells, compared to these cells in other rodent models, is an unique characteristic of GK rats [15] making the model valid also for studies of the peripheral nervous system. The relationship between ATF3 and cleaved caspase 3 stained Schwann cells and axonal outgrowth were evaluated in these rat models. The present nerve regeneration model utilizes the possibility of analysing ATF3 (i.e. activation) and cleaved caspase 3 (i.e. apoptosis) stained Schwann cells in relation to axonal outgrowth, where the cells at the lesion site (SNL) interact with the outgrowing axons, while the axons have not yet reached the analysis site in the distal nerve segment (SND; Figure 4; [9]).

Few studies have highlighted any gender differences or possible mechanisms in nerve regeneration (see e.g. [16–18]). Our results clearly show that there was a gender difference in axonal outgrowth after nerve injury and repair, with a longer outgrowth in male rats. The weight increase in the rats, a possible factor in a difference in axonal outgrowth, did not differ between male and female or even between healthy and diabetic rats and did not correlate with any of the variables; thus it could not explain the difference. However, our results are in accordance with previous studies using a nerve crush injury, i.e. a clinically less important injury [19], showing that neuroactive steroids, such as pregnenolone and progesterone metabolites that interact with progesterone receptor, are protective. This is also relevant for myelin repair in different neurological disorders [20]. Progesterone has been considered as an autocrine signalling molecule [21]. Interestingly, progesterone-loaded chitosan prostheses have been used to successfully reconstruct nerve injuries [22]; chitosan conduits being a promising clinical material for nerve reconstruction [23]. Potential use of neuroactive steroids, such as progesterone and testosterone, for treatment of diabetic neuropathy has also been suggested [5, 24], where differences between gender, diabetic state and a regional location, with request to neuroactive steroid levels, are reported [3, 25]. The reason(s) male subjects with diabetes develop neuropathy earlier than female subjects [1, 2] is not clear and may be complex, but factors such as testosterone deficiency, which is common in men with diabetes (i.e. leading to a pronounced deficit of neurosteroids), as well as lifestyle differences may contribute to the discrepancy in development, at least in type 2 diabetes in humans [26]. In addition, the mechanisms for the development of neuropathy in type 1 and 2 in humans may be different and particularly complex and multifactorial in type 2 diabetes [27, 28] which the present GK rat model resembles.

In our short-term model (i.e. six days), the regeneration process, which showed longer regeneration distances in male rats, may depend on the level of blood glucose. Thus, a moderately higher short-term blood glucose level may be advantageous for axons, leading to longer axonal outgrowth, while the opposite may be present in humans where higher long-standing blood glucose levels may have deleterious effects on the neurons and Schwann cells. An even more complex situation exists in diabetic BB rats, who have even higher blood glucose levels and in which axonal outgrowth does not differ from healthy rats in a short-term regeneration model (i.e. six days), although an increased number of activated and apoptotic Schwann cells are present after injury and repair in such BB rats [6]. The diabetic rat model used is crucial and gender aspects should also be considered in nerve regeneration studies. In long-term (i.e. three months) streptozotocin-induced diabetes in rats, the level of neuroactive steroids is lower than in healthy rats [3, 29, 30], which may contribute to explaining the findings of a shorter axonal outgrowth in experimental diabetes in the present study [3]. However, the pattern of various neuroactive steroid levels in the sciatic nerve among the healthy and diabetic rats in a study by Pesaresi et al. [3], who used another diabetes model, cannot be related to our findings regarding length of axonal outgrowth and numbers of ATF3 and cleaved caspase 3 stained Schwann cells. Thus, the neuroactive steroid levels may not entirely explain the differences between genders and state of glucose levels, but we cannot rule out the influence of neuroactive steroids since we did not measure such steroids. Moreover, several essential factors for nerve regeneration may be in play simultaneously.

Progesterone also promotes myelination, which we did not specifically address in the short-term experiments, by activating transcription factors (Krox-20) and/or myelin proteins (P0, PMP22; [21]). However, we did see gender differences, with higher numbers of ATF3 stained Schwann cells at the lesion site as well as in the distal nerve segment in male rats, which was particularly evident in the diabetic rats. The number of ATF3 stained Schwann cells correlated with axonal outgrowth, suggesting ATF3 has a role in axonal outgrowth [9], and it may even be suggested that ATF3 acts through different mechanisms in healthy and diabetic GK rats. ATF3, a member of CREB family, is a marker for activated Schwann cells and ATF3 stained Schwann cells and neurons rapidly increase locally as well as in dorsal root ganglia and in the spinal cord, respectively, after a nerve injury; this is an indication of cell viability [31]. In contrast, cleaved caspase 3 staining is a marker for apoptosis, i.e. cell death [32]. The numbers of ATF3 and cleaved caspase 3 stained cells correlated positively. Thus, the number of cleaved caspase 3 stained cells seems to balance the number of ATF3 stained cells at the lesion site in various models of diabetes [6] and in healthy rats. Such a mechanism has been suggested as a control function of Schwann cell proliferation, which is important [9, 33], although not crucial [34], for nerve regeneration.

The axonal outgrowth correlated with blood glucose levels when healthy and diabetic rats were analysed separately, which can probably be explained by the higher blood glucose levels in both healthy and diabetic male rats. In contrast to the present diabetic rat model, axonal outgrowth after nerve repair does not differ between female diabetic BB rats, which have much higher blood glucose levels, and female healthy rats [6]. However, these diabetic female rats also have an increased number of activated and apoptotic Schwann cells. Interestingly, the blood glucose levels in the present study correlated positively with the number of ATF3 stained Schwann cells in all rats and particularly in diabetic GK rats. The present higher numbers of cleaved caspase 3 stained Schwann cells in the diabetic female rats agrees with findings in diabetic BB rats [6] despite their different blood glucose profiles and potentially other dissimilarities. The blood glucose levels in diabetic rats correlated with both the numbers of ATF3 and cleaved caspase 3 stained Schwann cells; the latter finding was not surprising [35]. Apoptosis of Schwann cells and neurons is reported to be induced in STZ-induced diabetes and by glucose infusion due to oxidative stress [35, 36]. However, the diabetic BB model (i.e. female rats) had higher numbers of ATF3 stained cells, which contrasts with the present study, where female diabetic rats had fewer ATF3 stained cells than the healthy female rats [6]. There was also generally a lower number of DAPI stained cells in the distal nerve segment (Fisher p < 0.0001) in the diabetic rats, which agrees with findings in diabetic BB rats [6].

Not only is the blood glucose level per se relevant, but also the level of insulin, which may have a trophic support [37] making the use of different models relevant. In STZ-induced diabetes, the beta cells are destroyed by the toxic effect of STZ and in the BB rats autoimmune mechanisms that destroy beta cells are in action [38]. Any possible insulin effect is, therefore, mostly deleted in both such models, although there may be a lingering insulin production in STZ-induced diabetes [39], explaining why these rats survive without insulin supplementation over a period. In contrast, BB rats have no capacity to produce any insulin [39] and develop an insulin-dependent diabetes resembling that in humans. Such rats may also show symptoms similar to those of an autoimmune disease as well as pathological changes in the peripheral nervous system, which STZ-induced rats do not [40, 41]. Interestingly, there is no significant difference in the concentration of insulin between GK and Wistar rats [42]. Therefore, it is unlikely that the insulin level per se will explain the present changes in nerve regeneration. The influence of blood glucose levels on the nerve regeneration process is complex, as is also discussed when considering the development of diabetic neuropathy, where vibrotactile sense (i.e. sign of neuropathy) in patients with diabetes is not affected by the long-term blood glucose level, i.e. HbA1c, levels [43–45]. Heat Shock Protein 27 (HSP27) has recently been described tentatively as an important factor in the preservation of nerve function in patients with type 2 diabetes [46]. Whether HSP 27 is relevant in the present model, and not only in STZ-induced diabetes [47] or in BB/Wor rats with diabetes [48], remains to be investigated.

One limitation of our studies on nerve regeneration in diabetic GK and BB rats is the short-term design, which should be considered when interpreting nerve regeneration in diabetes and in connection with gender aspects. However, our approach was to relate axonal outgrowth with possible activation and apoptotic events concerning the Schwann cells in relation to certain blood glucose levels in male and female rats, without interfering with the processes in the treatment of insulin, which would be required in long-term studies in BB rats. Finally, the nature of the nerve injury is also relevant in this context, since a nerve compression lesion in diabetic rats induces a different response in the nerve trunk with more ATF-3 stained Schwann cells in diabetic BB rats than in diabetic GK and healthy rats [49]. Taken together, in future studies of nerve regeneration and neuropathy in diabetes, one may have to consider the diabetic model, the injury model and the gender of the rats as well as determine e.g. the levels of blood glucose, insulin, neuroactive steroids and potential protective substances, such as HSP27.

## Conclusions

Our study demonstrates gender differences in axonal outgrowth in diabetic GK and healthy rats after transection and repair after six days, with differences also detected in activation and apoptosis of Schwann cells in the sciatic nerve. This is interesting in view of the fact that males with diabetes have a higher frequency of neuropathy than females with diabetes and that males develop it more rapidly in humans [1, 2], indicating that relevant experimental models are compulsory. We suggest that it is extremely important to take into account gender differences as well as type of experimental diabetic model when new strategies and techniques for treatment of nerve injuries are developed.

## Methods

### Animals and surgery

Four groups of rats (initial body weight 200 g) were included: healthy male (n = 10) and female (n = 10) Wistar rats with normal blood glucose were compared with male (n = 10) and female (n = 10) Goto-Kakizaki rats (GK) in which a moderate increase in blood glucose is observed. Fasting blood glucose, body weight and polydipsia, as signs of diabetes, were measured and observed daily in all rats. Blood glucose was measured in a blood sample from the tail vein [Ascensia contour TM (Bio Healthcare, USA, Bio Diagnostics Europe) and LT (Bayer AB, Diabetes Care, Solna, Sweden); test slips (Microfil TM (Bio Healthcare Diabetes Care, USA)]. The rats had a 12 h day/night cycle in the cages and the GK rats were provided with extra water.

Before surgery the rats were anesthetized with intraperitoneal injection of a mixture of Rompun® (20 mg/ml; Bayer Health Care, Leverkusen, Germany) and Ketalar® (10 mg/ml, Pfizer, Helsinki, Finland) at a dose of a 1 ml Ketalar® and 0.25 ml Rompun® per 100 g body weight of the rat. Postoperatively, all rats were treated with Temgesic® 0.01-0.05 mg/kg (0.3 mg/ml; Schering-Plough Europe, Brussels, Belgien) to prevent pain. The sciatic nerve in the hind limb in each rat was unilaterally exposed, transected and instantly repaired with 9-0 ethilon (Ethicon®) epi/perineurial sutures (Figure 4). The muscles were secured with resorbable sutures, the skin was closed and the rats were allowed to recover.

Six days after surgery the rats were killed by an overdose of pure pentobarbitalnatrium (60 mg/ml; Apoteksbolaget, Malmö, Sweden). The sciatic nerve was harvested bilaterally. The samples were fixed in Stefanini solution [4% paraformaldehyde and 1.9% picric acid in 0.1 M phosphate buffer (PBS) pH 7.2] for 24 hours. After the fixation procedure the samples were washed (PBS × 3) and placed in 20% sucrose solution over night. Before cryostat sectioning the samples were placed in OCT Cry mount® (Histolab products AB, Gothenburg, Sweden) for embedding. After freezing the samples the nerves were sectioned longitudinally at thickness of 8 μm on superfrost plus glass (Thermo scientific, Braunschweig, Germany).

### Immunohistochemistry

After washing (PBS 5 min) the sections were incubated with anti-human neurofilament protein (DAKO Glostrup, Denmark; dilution 1:80 in 0.25% Triton-X 100 and 0.25% BSA; bovine serum albumin) in PBS, over night at 4°C. On day two, after additional washing (PBS 3×5 min), the slides were incubated with the second antibody Alexa Fluor 594 conjugated goat anti-mouse IgG (Invitrogen, Molecular Probes, Eugene, Oregon, USA; 1:500 in PBS) for one hour, at room temperature. The slides were then washed (PBS 3×10 min), counterstained with 4’,6’-diamino-2-phenylindole DAPI (Vectashield®, Vector Laboratories, Inc. Burlingame, CA 94010, USA) to visualize the nuclei (i.e. for counting the total number of cells) and then mounted and cover slipped.

Other sections were instead incubated with rabbit anti-ATF-3 polyclonal antibody (Santa Cruz Biotechnology, USA; dilution 1:200) as the primary antibody and Alexa Fluor 488 conjugated goat anti-rabbit IgG (Invitrogen, Molecular Probes, Eugene, Oregon, USA; dilution 1:500) as the secondary antibody.

In further sections the antibody against cleaved caspase 3 (BioNordica, Stockholm, Sweden, dilution 1:200) together with the secondary antibody Alexa Fluor 488 conjugated goat anti-rabbit IgG (Invitrogen, Molecular Probes, Eugene, Oregon, USA; dilution 1:500) was used to count the apoptotic cells.

Double immunohistochemical staining (S-100 and ATF-3) was done to ensure that only Schwann cells were counted. The slides were incubated day one with rabbit anti-ATF-3 polyclonal antibody (Santa Cruz Biotechnology, USA, dilution 1:200) and on day two with the secondary antibody Alexa Fluor 488 conjugated goat anti-rabbit IgG (Invitrogen, Molecular Probes, Eugene, Oregon, USA; dilution in 1:500). After one hour the slides were incubated with mouse monoclonal IgG anti-S100 antibody α/β chain (Santa Cruz Biotechnology, USA; dilution 1:200). On day three, the sections were incubated with Alexa Fluor 594 conjugated goat anti-mouse IgG (Invitrogen, Molecular Probes, Eugene, Oregon, USA; dilution1:500) and one hour later mounted with VECTASHIELD (Vectashield®, Vector Laboratories, Inc. Burlingame, CA 94010, USA) and cover slipped. A similar double immunohistochemistry procedure was used for staining of cleaved caspase 3 stained Schwann cells (i.e. S-100).

### Photography and computer analysis

Sections from each nerve were blind coded before the analyses in the digital system. From the contralateral uninjured side only a few sections were analyzed, but not quantified, to see whether there was any difference between healthy female and male and GK diabetic female and male rats. The sections were photographed using a fluorescence microscope provided with a digital system camera (Nikon 80i) system connected to a computer. The digital images were analysed using the NIS elements computer program. The length of the stained neurofilament proteins was measured in three randomly selected sections (mean value from the three sections; Figure 4) from the lesion site to the front of the longest growing axons [6]. The stained cells for ATF-3 and cleaved caspase 3 stained cells were counted in three sections (image size 500 μm ×400 μm; mean values from three sections) from the sciatic nerve both at the lesion site (SNL) and in the distal nerve segment (SND) beyond the stained neurofilaments (i.e. at 8 mm from nerve lesion) [9]. At these locations, squares (6 × 100 μm^2^) were randomly selected and examined for the presence of cleaved caspase 3 and ATF-3 stained Schwann cells (Figure 4). Exactly the same squares were also used to count the total number of DAPI stained cells.

### Statistical methods

IBM SPSS Statistics version 20 was used for the statistical analysis. The results are presented as median values [with 25^th^ - 75^th^ percentiles] since the data were not considered to be normally distributed and thus required non-parametric tests for further analyses. To detect any significant values among the four groups, the non-parametric method Kruskal-Wallis (KW) was used and with the post hoc Mann-Whitney (MW) test to observe differences between the following groups: healthy/diabetic and male/female. Separate p-values for males and females were then combined in to an overall p-value by using the Fisher method for independent samples based on the chi square distribution [50] (http://en.wikipedia.org/wiki/Fisher's_method, courtesy of our statistician, Professor Jonas Björk, Lund University, Lund, Sweden). A p-value of less than 0.05 was regarded as significant.

### Ethics

The animal ethics committees in Malmö and Lund approved all animal experiments (Lund University permit number: 347/2011). The study was carried out in strict accordance with the recommendations made by the Swedish Board of Agriculture and the European Union. All efforts were made to minimize suffering.

## Abbreviations

BB: Biobreeding
ATF3: Activation Transcription Factor 3
GK: Goto-Kakizaki
PBS: Phosphate Buffered Saline
MW: Mann-Whitney
KW: Kruskal-Wallis
P0: Protein zero
PMP22: Peripheral Myelin Protein 22
CREB: CAMP Response Element Binding
STZ: Streptozotocin
HbA1c: Haemoglobin A1c
HSP27: Heat Shock Protein 27
BB/Wor: Biobreeding/Worchester.
