# Correction to: Gender differences in nerve regeneration after sciatic nerve injury and repair in healthy and in type 2 diabetic Goto-Kakizaki rats

**DOI:** 10.1186/s12868-020-00594-0

**Published:** 2020-11-03

**Authors:** Lena Stenberg, Lars B. Dahlin

**Affiliations:** Department of Clinical Sciences - Hand Surgery, Lund University, Skane University Hospital, Malmö, Sweden

## Correction to: BMC Neurosci 15:107 (2014) https://doi.org/10.1186/1471-2202-15-107

Following the publication of the original article [1], the authors reported an error in the calculation of the total cell number at SNL and SND.

The values for the total cell number (mm^2^) SNL and total cell number (mm^2^) SND in Table 1 (page 2) should be multiplied by 4.7. This does not change anything about the other results or the drawn conclusion in the paper.

**Table 1:** Nerve regeneration after nerve injury and repair in healthy and diabetic rats

	Healthy Male (n = 10)	Healthy Female (n = 10)	Diabetes Male (n = 10)	Diabetes Female (n = 10)	p-values (KW^a^)	Fisher’s method^b^	
						Male/ female	Healthy/ Diabetes
Axonal outgrowth (neurofilaments, mm)	7.0 (6.8–7.3)	6.3 (6.1–6.4)	6.9 (6.7–7.1)	5.8 (5.1–6.2)	*0.0001*	< *0.0001*	*0.02*
ATF-3 (% of total) SNL	17.8 (17.6–18.4)	17.6 (16.7–18.0)	19.8 (19.1–21.1)	16.4 (15.7–17.3)	*0.0001*	*0.0001*	*0.001*
ATF-3 (% of total) SND	19.2 (17.7–19.6)	19.5 (18.9–20.1)	18.7 (16.9–20.1)	15.2 (14.4–15.7)	*0.0001*	*0.0003*	*0.001*
Cleaved caspase-3 (% of total) SNL	5.2 (2.6–7.8)	3.1 (2.9–3.4)	9.6 (8.7–11.6)	7.0 (6.4–10.1)	*0.0001*	0.31	< *0.0001*
Cleaved caspase-3 (% of total) SND	2.7 (2.2–4.0)	2.5 (2.4–2.7)	6.4 (4.5–7.1)	5.8 (4.3–6.1)	*0.0001*	0.39	< *0.0001*
Total cell number (mm^2^) SNL	5344 (5297–5367)	4935 (4827–5010)	5043 (4916–5184)	5118 (4977–5161)	*0.0001*	*0.0005*	*0.0001*
Total cell number (mm^2^) SND	5344 (5273–5414)	5226 (5020–5391)	4949 (4789–5128)	5137 (4911–5179)	*0.0001*	0.07	< *0.0001*
Preoperative B-glucose (mmol/l)	4.6 (4.3–4.9)	3.90 (3.8–4.3)	15.0 (13.3–17.2)	10.3 (9.7–11.7)	*0.0001*	< *0.0001*	< *0.0001*
Weight increase (%)	2.2 (0.7–2.7)	2.4 (1.3–4.8)	2.0 (1.2–2.6)	2.6 (2.0–3.2)	0.34	–	–

Values are median 25th (Q1)−75th (Q3) percentiles. p-values in italics is significantly different at least at the 0.05 level. ^a^KW = Kruskal–Wallis. ^b^Fisher method for independent samples based on the chi square distribution.* SNL* site of lesion, *SND* distal nerve segment. Total cell = DAPI stained cells

The amended version of Table 1 can be found below.
